# Supplementary material for: Draft Sequencing Crested Wheatgrass Chromosomes Identified Evolutionary Structural Changes and Genes and Facilitated the Development of SSR Markers
Source: Int J Mol Sci. 2022 Mar 16;23(6):3191. doi: 10.3390/ijms23063191 (PMC8948999; doi:10.3390/ijms23063191)
Supplement: Supplementary file 1 [file ijms-23-03191-s001.zip › TableS2.pdf]

Table S2: The most frequent genes annotated in the draft assemblies of *Agropyron cristatum*.

Chromosome	Frequency	Annotated genes
1P	6	Protein phosphatase PP2A regulatory subunit B
	4	Arginine--tRNA ligase | Phosphomethylpyrimidine synthase
	3	30S ribosomal protein S3 | CTP synthase | Enolase | Formin-like protein 3 | Kinesin-like protein KIN-14K | Ornithine aminotransferase | Polyamine oxidase 5 | Protein RecA | Senescence-specific cysteine protease SAG39 | Succinate--CoA ligase [ADP-forming] subunit beta | Superoxide dismutase [Mn] | Thymidylate synthase | Uroporphyrinogen decarboxylase | Very-long-chain aldehyde decarbonylase GL1-7 | V-type proton ATPase subunit D
2P	9	Amidase 1
	8	Deoxyuridine 5'-triphosphate nucleotidohydrolase | Nuclear/nucleolar GTPase 2
	6	Germin-like protein 8-5
	5	Cortical cell-delineating protein | Ent-kaur-16-ene synthase | Non-specific lipid-transfer protein 2G | Probable nicotianamine synthase 2
3P	16	Glucan endo-1
	14	Rust resistance kinase Lr10
	13	Putative glutaredoxin-C2
	11	Protein DETOXIFICATION 40
	10	Cationic peroxidase SPC4
	9	16.9 kDa class I heat shock protein 1
	8	ABC transporter B family member 20 | Amino-acid permease BAT1 homolog | Sugar transport protein MST3

Chromosome	Frequency	Annotated genes
	7	Abscisic stress-ripening protein 1 | Germin-like protein 1-3 | Germin-like protein 1-4 | Zinc finger protein KNUCKLES
4P	4	Fructose-1 | Glutamate--tRNA ligase | Oxygen-dependent coproporphyrinogen-III oxidase | Phosphoenolpyruvate carboxykinase (ATP)
	3	Adenylate kinase | Bifunctional purine biosynthesis protein | Elongation factor G | Glyceraldehyde-3-phosphate dehydrogenase | H/ACA ribonucleoprotein complex subunit CBF5 | Isoleucine--tRNA ligase | Malate dehydrogenase | Multidrug resistance protein CDR1 | Ornithine aminotransferase | Phosphoribosylaminoimidazole carboxylase | Probable peptidyl-prolyl cis-trans isomerase | Serine hydroxymethyltransferase | Transaldolase
5P	15	Putative ripening-related protein 4
	9	Probable aldo-keto reductase 2
	8	DEAD-box ATP-dependent RNA helicase 38 | Deoxyuridine 5'-triphosphate nucleotidohydrolase
	7	ATP synthase subunit alpha | DEAD-box ATP-dependent RNA helicase 1 | Membrane protein PM19L
	6	ATP-dependent zinc metalloprotease FTSH 4 | Auxin-responsive protein SAUR36 | Carotenoid 9 | Histone H4 | Phenylalanine ammonia-lyase | Tryptophan aminotransferase-related protein 1
6P	7	ATP synthase subunit alpha | High-affinity nitrate transporter 2.2 | S-(+)-linalool synthase
	6	Callose synthase 3 | Histone H2B.2 | Histone H4 | Senescence-specific cysteine protease SAG39
	5	Bidirectional sugar transporter SWEET14 | Cinnamoyl-CoA reductase 1 | DExH-box ATP-dependent RNA helicase DExH12 | L-arabinokinase | Probable O-methyltransferase 2 | Protein H2A.6
7P	12	ABC transporter C family member 10
	5	Guanine nucleotide-binding protein alpha-1 subunit
	4	Callose synthase 10 | Replication factor C subunit 1
	3	ABC transporter B family member 20 | Auxin response factor 12 | Callose synthase 5 | Exopolygalacturonase | Kinesin-like protein KIN-14L | Kinesin-like protein KIN-UB | Leucine--tRNA ligase | MADS-box transcription factor 27 | Pre-

Chromosome	Frequency	Annotated genes
		mRNA-splicing factor ATP-dependent RNA helicase DEAH1 | Probable metal-nicotianamine transporter YSL9 | Protein translocase subunit SecA | Transcription initiation factor TFIID subunit 5
