# Supplementary figures and images for: Draft Sequencing Crested Wheatgrass Chromosomes Identified Evolutionary Structural Changes and Genes and Facilitated the Development of SSR Markers
Source: Int J Mol Sci. 2022 Mar 16;23(6):3191. doi: 10.3390/ijms23063191 (PMC8948999; doi:10.3390/ijms23063191)

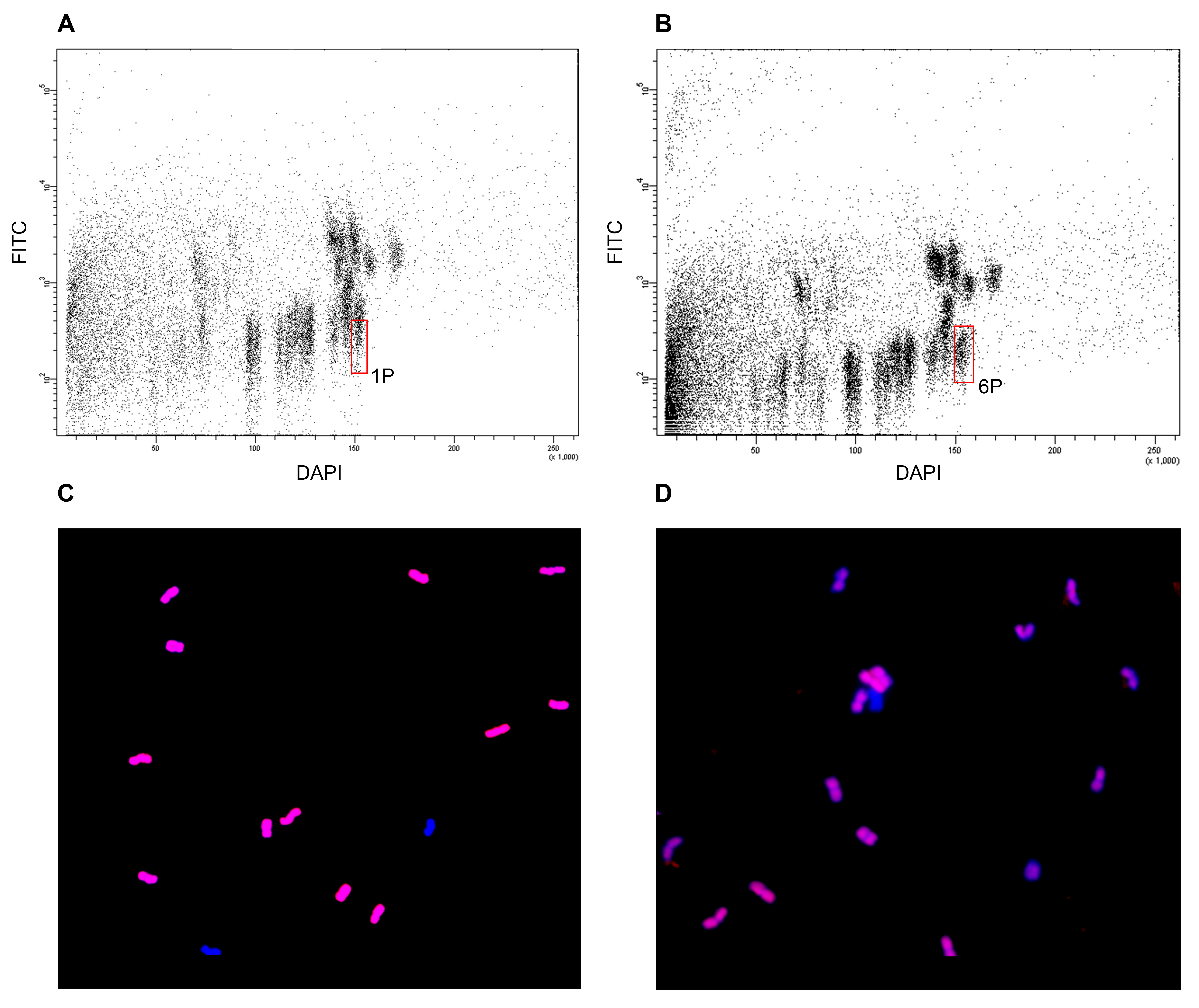

Supplement: Supplementary file 1 [file ijms-23-03191-s001.zip › FigureS1.tif]

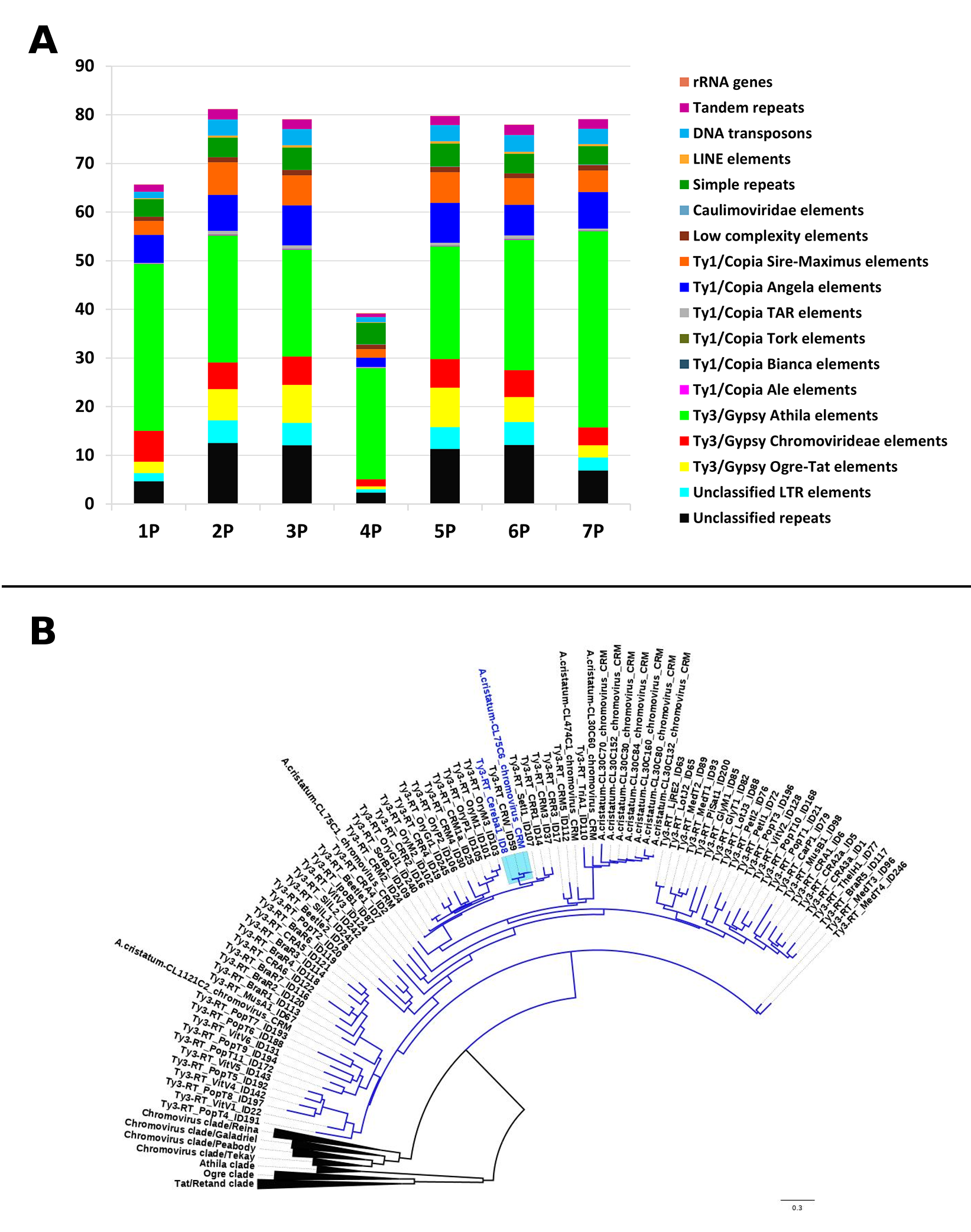

Supplement: Supplementary file 1 [file ijms-23-03191-s001.zip › FigureS2.tif]
